# Mechanical Properties of Glass Ionomer Cements after Incorporation of Marine Derived Hydroxyapatite

**DOI:** 10.3390/ma13163542

**Published:** 2020-08-11

**Authors:** Maja Bilić-Prcić, Valentina Brzović Rajić, Ana Ivanišević, Ana Pilipović, Sevil Gurgan, Ivana Miletić

**Affiliations:** 1 School of Dental Medicine, University of Zagreb, Gundulićeva 5, 10000 Zagreb, Croatia; bilicprcic.maja@gmail.com (M.B.-P.); vbrzovic.rajic@sfzg.hr (V.B.R.); miletic@sfzg.hr (I.M.); 2 Faculty of Mechanical Engineering and Naval Architecture, University of Zagreb, Lučićeva 5, 10000 Zagreb, Croatia; ana.pilipovic@fsb.hr; 3 School of Dentistry, Hacettepe University, Ankara 06100, Turkey; sgurgan@gmail.com

**Keywords:** glass ionomer cement, mechanical properties, hydroxyapatite

## Abstract

The purpose of this study was to evaluate the effects of the incorporation of hydroxyapatite (HA) derived from cuttlefish bone on the mechanical properties of glass ionomer cements (GIC). Fuji II LC and Fuji IX GP Extra (GC Corporation, Tokyo, Japan) were used in the study. There were four groups (*n* = 11–18) for each material: a group without the addition of HA particles and three groups modified by incorporation of 2, 5, and 10 wt% HA. The tests were performed on a universal testing machine (Shimadzu, Duisburg, Germany) and descriptive statistics, two-way analysis of variance (ANOVA) for the comparison of three mechanical properties, and one-way ANOVA for the comparison of different concentrations for each material were performed. Regarding the Fuji IX groups, compressive strength (CS) and flexural strength (FS) were highest in the group without HA particles added. The differences in CS between the Fuji IX group without HA particles and the Fuji IX groups with 2 wt% HA and 10 wt% HA were significant. The Fuji II 5 wt% HA group exhibited higher diametral tensile strength (DTS) and CS than other Fuji II groups, but not significantly. The Fuji II group, modified with 10 wt% HA, exhibited significantly higher FS than the Fuji II group without HA particles (*p* < 0.05). Porous HA incorporated into the Fuji IX groups had a significant impact on mechanical properties only in the Fuji IX 5 wt% HA group. Fuji II groups modified with 10 wt% HA showed the most favorable results with respect to FS.

## 1. Introduction

Glass polyalkenoate cements, also called glass ionomer cements (GIC), are materials made of calcium or strontium aluminum fluorosilicate glass powder and a soluble polymer. The two components go through an acid-base setting reaction in the presence of water [1]. The main advantages of these materials are their biocompatibility, their anticariogenic and remineralization effects, and their ability to chemically bond to calcified dental tissues [1,2]. Major shortcomings of GICs as permanent posterior and anterior restorations include insufficient mechanical properties including compressive strength (CS), hardness, fracture toughness, elastic modulus, and wear resistance [1,3].

To improve their poor mechanical properties, GICs have undergone numerous modifications since their invention 50 years ago by Wilson and Kent [4]. In this aspect, better physical properties have been achieved by optimizing the powder/liquid (p/L) ratio and particle size and distribution [5]. Owing to a high p/L ratio and reduced glass particle size, Fuji IX GP Extra is a highly viscous material [6], and some studies have confirmed that high-viscosity GICs have superior physical properties as compared with conventional GICs [7]. Furthermore, resin-modified (RM) GICs are light-cured materials with better mechanical properties than conventional GICs [8]. They contain the same essential components as conventional glass ionomers (base, acid, water), but they also include a monomer, typically 2-hydroxyethyl methacrylate (HEMA), and an associated initiator system, camphorquinone [8].

Moreover, many studies have shown that the incorporation of various fillers into cement powder, including stainless steel, silver, zirconia, zinc, carbon and aluminosilicate fibers, cellulose, titanium dioxide, hydroxyapatite (HA) and others, have resulted in improved mechanical properties [9,10,11,12,13,14,15,16,17].

Recently, it was shown that HA has promising advantages in restorative dentistry. HA is a biologically developed apatite, which is a major calcified component of tooth and bone. It was reported that the addition of HA increased the hardness of GICs, and HA was found to interact with carboxylate groups in the polyacids of GICs [18]. Furthermore, spherical porous HA particles were most effective in remineralisation and increasing mechanical and antibacterial properties [19]. The endeavors of preparing synthetic materials with suitable structure and biocompatibility led to the use of HA from natural sources like cuttlefish bone in medical and dental applications [20]. Highly porous HA can be prepared from aragonitic cuttlefish bone by using the hydrothermal method [21].

Marine-derived HA and collagen have recently been recognized as promising biomaterials in other fields of medicine, especially bone tissue engineering [22,23]. It was reported that marine-derived scaffolds containing nano HA used in craniofacial bone reconstruction and regenerative procedures resulted in favorable clinical outcomes due to good adhesion of mesenchymal stem cells, proliferation, and osteogenic differentiation [22]. Besides being biocompatible, marine-derived biomaterial is becoming increasingly attractive due to its low cost and availability [22].

The outcome of this study should highlight the effect of the addition of cuttlefish bone-derived HA particles in different wt% to Fuji IX GP Extra and Fuji II LC on their mechanical properties, including CS, flexural strength (FS), and diametral tensile strength (DTS). The improvement of the mechanical properties of GIC based materials is of significant clinical importance since GIC materials are more biocompatible than dental resins, but their mechanical properties are relatively weaker [1,3,6]. Moreover, it was previously reported that HA incorporated into GIC improved the strength of GICs, but it also increased the release of fluoride ions contributing to anticariogenic properties of these materials, which is clinically truly relevant [24,25].

The null hypothesis was that the GICs modified with HA would not show significantly better performance in terms of mechanical properties than the materials without HA added to the powders of two commercially available GICs.

## 2. Materials and Methods

Cuttlefish bones (Sepia officinalis L.) from the Adriatic Sea were used for the hydrothermal synthesis of HA, as previously described [21].

Two commercially available GICs were used in this study: Fuji II LC, shade A2, and Fuji IX GP Extra (GC Corporation, Tokyo, Japan). Cuttlefish bone-derived HA was ground and sifted through a 180 μm size sieve leaving the HA powder in the form of hexagonal column crystal aggregates with a diameter of <180 μm. The fluoro-alumino-silicate glass powder and HA powder were hand-mixed with a mortar and pestle for 20 min to obtain as uniform a distribution of HA as possible. The prepared powder was then mixed with the polyacid liquid by spatulation. Four groups were prepared for each GIC material. There were not any HA particles added in the first group, while the powder in the other three groups was modified by incorporation of 2, 5, and 10 wt% HA, respectively. The recommended powder-liquid (p/L) ratio could not be obtained because of the high bulk density of HA powder [16]. For Fuji IX groups, the p/L ratio was 2, and for the Fuji II groups, the p/L ratio was 3.

For CS testing, cylinder-shaped specimens were prepared for each group (N = 16–18). After mixing, the material was poured into a syringe (Centrix, Shelton, CT, USA) and immediately into silicone moulds (4 mm diameter and 8 mm height). To avoid air trapping, polyester strips were placed, and the material was gently compressed on both sides of the mould. Fuji IX specimens were left for one hour to allow the material to set. Fuji II specimens were irradiated from both sides of the mould, 40 s per side by using a LED lamp (Ivoclar Vivadent AG, Schaan, Lichtenstein, Germany), with intensity 600 mW/cm^2^. The specimens were carefully removed from the mould 1 h after irradiation and stored in deionized water for 7 days before polishing and testing. The excess cement was removed by polishing both sides of the steel mould of the right dimensions, 4 mm diameter and 6 mm height, with 500-grit carbide paper under continuous water irrigation on a grinder-polisher (Buehler, IL, USA). Some specimens were accidentally destroyed during this procedure, so they were discarded from further testing. CS was performed according to ISO 9917-1:2007 [26] at a speed of 0.75 mm/min, room temperature of 22 °C, and relative humidity of 45%. The CS, measured in N/mm^2^, for each specimen was calculated by using the equation:

(1) $$CS=\frac{4\cdot F}{\pi \cdot d^{2}}$$

where *F* (N) is the force at fracture and *d* (mm) is the diameter of the specimen. Mean values and standard deviations were determined for each group.

For the DTS measurement, cylindrical specimens were prepared by using a split silicone mold (5 mm high and 6 mm in diameter), according ANSI/ADA Standard No. 27 [27]. The specimens were then polished to 4 mm × 6 mm in steel moulds. There were 15–18 specimens in each group, and their preparation was the same as for CS testing. During the measurements, compressive force was applied along the diameter of each sample at a speed of 0.5 mm/min. The maximum force applied was used to calculate the DTS by using the equation:

(2) $$DTS=\frac{2\cdot F}{\pi \cdot d\cdot l}$$

In which DTS (N/mm^2^) is diametral tensile strength, *F* (N) is fracture load, *d* (mm) is the diameter of the specimens, and *l* (mm) is the length of the specimens.

For FS measurements, specimens were prepared by using a split silicone mould with internal dimensions of 25 mm × 2 mm × 3 mm, which was polished to a right dimension of 25 mm × 2 mm × 2 mm, according to ISO 9917-2:2007 standards [26]. There were between 11 and 17 specimens in each group, and they were prepared the same as specimens for CS and DTS testing. The bars were light-cured with a series of three 20 s irradiations in the middle and at each end of each sample. FS measurements were performed according to ISO 9917-2:2017 standard [28], with a speed of 0.75 mm/min. FS was calculated by using the equation:

(3) $$\sigma =\frac{3\cdot F\cdot L}{2\cdot b\cdot h^{2}}$$

In which σ (N/mm^2^) is flexural strength, *F* (N) is maximum force, *L* (mm) is the distance between the supports (*L* = 20 mm), *b* (mm) is width and *h* (mm) is the height of the test specimen.

**Figure 1:**
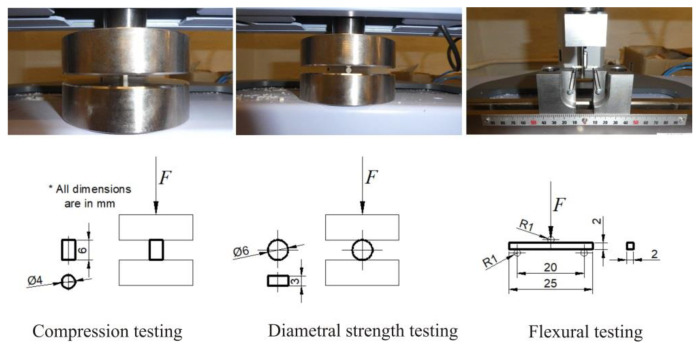
Schematic and actual testing of compression, diametral and flexural properties.

Figure 1 shows the test procedures for all three properties—compression, diametral, and flexural (CS, DTS, and FS).

All mechanical properties were tested on a universal testing machine (Shimadzu, Duisburg, Germany) with a maximum cell load of 10 kN. Regarding a statistical analysis of the data, a descriptive analysis, a two-way ANOVA and a post-hoc Tukey’s test were performed. Distribution normality was tested with the Shapiro-Wilk test and equality of variances was tested with Levene’s test. The analysis was performed using a SAS statistical package on a Windows platform. The level of significance was set at *p* = 0.05.

## 3. Results

Descriptive statistics for the eight groups (Fuji II and Fuji IX groups) and the three mechanical properties are shown in Table 1, Table 2 and Table 3.

**Figure 2:**
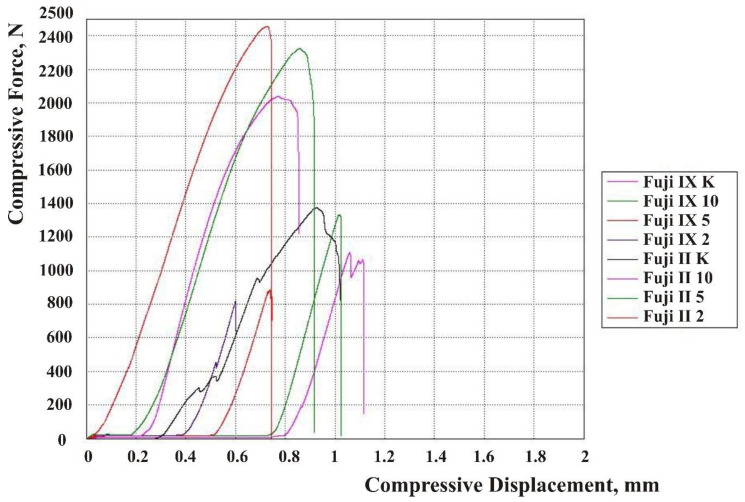
Compressive force-displacement diagram for the Fuji IX and Fuji II groups.

**Figure 3:**
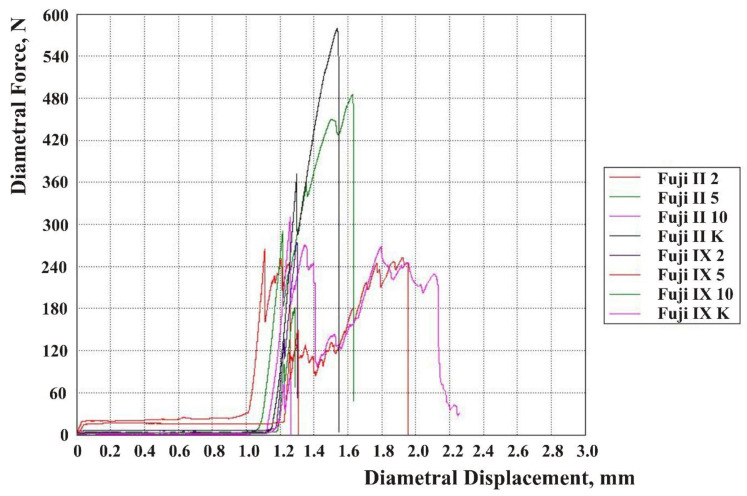
Diametral tensile force-displacement diagram for the Fuji IX and Fuji II groups.

**Figure 4:**
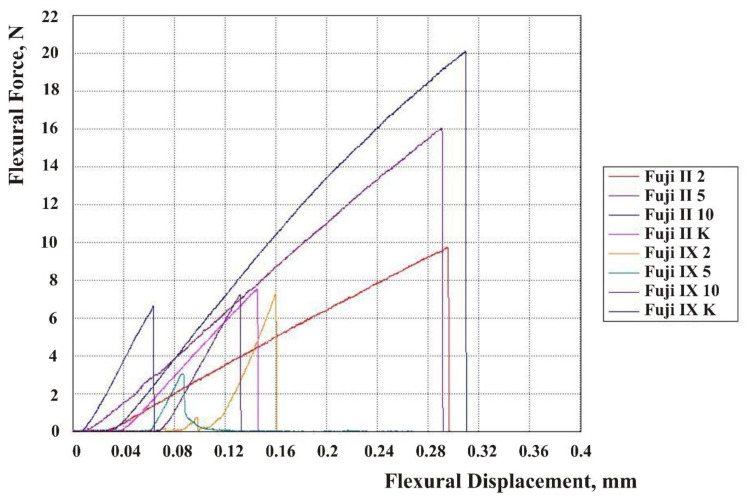
Flexural force-displacement diagram for the Fuji IX and Fuji II groups.

The results are shown in force-displacement diagrams for CS, DTS, and FS Figure 2, Figure 3 and Figure 4. Each line in the diagram represents the mean of one sample group (Fuji IX and Fuji II with 2, 5, and 10 wt% HA and without HA). Despite mold production of specimens and subsequent polishing, not every surface was ideally parallel, and no prestressing was applied when placed in the jaw of the testing machine. The upper jaw of the machine did not fully touch the surface of each specimen. For this reason, the curves on the diagrams were flat at the beginning of the test. When the test started, a force exerted on a specimen’s surface was very small because only a small area of the surface was engaged.

CS, DTS, and FS data follow normal distribution (Shapiro-Wilk test). There was no difference in standard deviations between eight groups for all three mechanical properties. Results of a two-way ANOVA test for comparison of three mechanical properties are presented in Table 4.

Comparison between different groups regarding CS, DTS, and FS showed that there were differences between groups (*p* < 0.0001; two-way ANOVA test). However, there was no significant difference in DTS between the Fuji II group without HA and Fuji II groups modified with 2 wt%, 5 wt%, and 10 wt% HA. There was also no significant difference in DTS between the Fuji IX group without HA and Fuji IX groups modified with 2 wt%, 5 wt%, and 10 wt% HA. Since the interaction between material and HA wt% was significant for CS, a separate analysis was performed to detect differences between the groups. Comparison of different concentrations for each material by one-way ANOVA revealed that there is no difference between four Fuji II groups (Table 5). However, the CS of the Fuji IX groups modified with 2 wt% HA and with 10% HA were significantly lower than CS of the Fuji IX group without HA (one-way ANOVA and Tukey test, Table 6).

The interaction between material and HA concentration was also significant for FS. The FS of the Fuji II group modified with 10 wt% HA was significantly higher than the FS of the Fuji II group without HA, but the difference was not significant compared to the Fuji II groups 2 wt% and 5 wt% HA. The FS of the Fuji IX group modified with 5 wt% HA was significantly higher than the FS of the Fuji IX group without HA.

## 4. Discussion

Despite the number of advantages of GIC-based materials used in restorative dentistry, their main disadvantages have to do with inadequate mechanical properties. This fact has prompted many studies in which commercially available cements have been modified with different microparticles and nanoparticles, including micro- and nano-sized HA particles, in an attempt to obtain improved mechanical properties and clinical performance [9,13,29]. Microparticles of HA have been shown to be easily mixed with GIC powder, including resin [22], while porous spherical HA particles have been shown to increase mechanical properties and the release of fluoride ions most effectively [19,30]. According to the literature, there are no data about the effect of porous spherical HA particles derived from cuttlefish bone on the mechanical properties of chemically-set GICs and light curing GICs. The purpose of this study was to determine the effect of the incorporation of porous HA particles obtained from cuttlefish bone (< 180 µm) in different wt% on the CS, DTS, and FS of light-curing and chemically-set GICs.

The recorded improved mechanical properties of the Fuji II group after incorporating 10 wt% HA and associated improvements in FS probably resulted from the ‘umbrella phenomenon of resin polymer’ that protects the calcium polyacrylates from dissociation in the early phase of the setting, before maturation and the formation of more stable aluminum polyacrylates [1]. It was indeed shown that resin-modified GICs are more stable in a wet and acidic environment than are conventional GICs [31,32]. However, unlike conventional GICs, restorative materials containing resin have been shown to have negative effects on dental pulp; they also exhibit cytotoxicity, and they prompt biofilm formation and secondary caries development [33]. It would therefore be ideal in a clinical context to obtain better mechanical properties of chemically set GICs. This research did not yield any result that could be used for the improvement of mechanical properties of chemically set GIC by incorporating HA particles into fluoro-alumino-silicate glass powder.

In fact, adding HA particles to Fuji IX powder resulted in reduced CS, DTS, and FS when compared to the samples without HA. Although the absolute values of the three mechanical properties recorded cannot be compared with the values in other studies due to different experimental settings, we can notice that the trend of reduced CS after the addition of HA particles in the present study is not in agreement with the results of a study by Alatawi et al. [15], which indicated that GICs with different quantities of incorporated nanoHA resulted in higher CS values. It could be that the incorporated particles in the present study interfered with the reaction between the modified powder and liquid, thus reducing polysalt formation, since the HA particles used in this study were relatively large, the surface area was smaller, and the quantity of released ions that participated in the acid-base reaction between polyacrylic acid and powder was relatively lower [17,34]. On the other hand, in a study by Alatawi et al. [15], nanoHA could participate in each GIC polysalt bridge formation during setting, leading to improved mechanical properties of the cements [27,35]. Besides, the trend of reduced values in this study could be assigned to the soaking of the specimens in distilled water for a week, which could lead to a gradual degradation of bonds between HA particles and the GIC matrix [10]. After the initial setting of chemically set GICs, such as Fuji IX, the setting reaction continues for a few days in a maturation process. During this time, aluminum salt bridges form, which influences the final mechanical properties of the cements. In this phase, the degree of cross-linking between polyacrylic acid and incorporated HA particles probably also increases [36]. The ratio of the total leachable ionic ratio of Ca/Al is increased in favor of Ca in HA containing formulas when the glass-ionomer structure is known to be stabilized by the trivalent Al crosslinking with polyacrylic acid. Another reason for the reduced mechanical properties in the HA-modified Fuji IX samples in this study could be the fracturing of HA particles since their size was too big, < 180 microns, and HA particles of a size of 10–20 microns were shown to have a better effect on improving mechanical properties [30].

The liquid-to-powder ratio in the present study was lower than recommended by the manufacturer, and for this reason, the values obtained in the study cannot be compared with the values reported in previous studies, as already mentioned. The modified powder saturated the liquid component of GIC at a lower *p*/L ratio, and the recommended ratio of 3.6 could not be achieved due to the oversaturation of liquid and poor interfacial bonding. The *p*/L ratio for the Fuji IX groups with modified powder was 2, and was the same in the group without HA particles added, so the powder modification would be the only variable.

On the other hand, the incorporation of HA particles into Fuji II powder had a positive effect on mechanical properties, and the group in which the powder was modified with 10 wt% HA resulted in a significantly higher FS value. The improvement of CS, FS, and DTS after modification of Fuji II powder with apatite particles is consistent with previous findings [37]. One could, however, argue that these results could not be compared to the results of the present study since the particles added to GIC powder were < 180 µm and not of nano size. However, a study by Arita et al. [30], which focused on the influence of characteristics of HA particles on mechanical properties of modified conventional GICs, an SEM analysis showed that the particle size of nano-HA was about 10–20 nm, but the crystals clustered together to form particles greater than 10 µm in diameter. Also, HAp100 particles used in the same study [30] comprised columnar crystals about 200–300 nm in diameter, but it condensed into aggregates of 200–300 µm. Therefore, it is important to consider the size of crystal clusters when evaluating the mechanical properties of GICs. The particles in our study were obtained by hydrothermal conversion of cuttlefish bone into HA, during which its original microstructure was preserved. The HA particles formed by hydrothermal conversion have a cauliflower-like morphology, thus increasing the surface roughness and specific surface energy [21]. Moreover, it was shown that the difference in microleakage was not significantly different between the modified cements with incorporated nano- and micro-HA particles, implying that particle size cannot significantly affect the reaction [38].

Mechanical properties indicate the resistance of a material to deformation and fracture under a force applied to the material’s surface unit area. The strength of a brittle material such as GIC is the level of stress at which fracture occurs, and dependent on the direction of the force applied, it is expressed in terms of CS, FS, and DTS. This is particularly important for the dentist in planning a restoration or estimating resistance to cracking of a restorative material under masticatory stress. In their paper, Marelli et al. [39] analysed mechanical strength and microhardness of zirconia. Interestingly, the authors observed a strong effect of surface roughness on the flexural strength of zirconia samples, and surface polishing caused a strong increase in the average values of flexural strength [39]. Since the surfaces of the samples in the present study were all prepared in the same manner, we can conclude that the observed difference in flexural strength, especially significantly improved FS in the case of Fuji II modified with 10wt% HA, is exclusively the result of the addition of HA particles.

Limitations of this study are concerned with samples preparation, primarily the manual spatulation resulting in possible air inclusions, and inability to achieve the liquid-to-powder ratio recommended by the manufacturer due to the nature of the marine-derived micro HA particles incorporated into the powders of Fuji II and Fuji IX GIC materials. It would be of great clinical interest in further research to study the effect of HA particles incorporated into GIC material on the demineralizing capacity of the material. Furthermore, dental pulp stem cells, as well as other oral derived stem cells, represent easily accessible and free of ethical dilemmas source of cells for use in regenerative medicine [40,41,42]. It is particularly important to avoid any immunological reaction to the xenogenic material in the tissues from which the stem cells should be recovered and used in regenerative medicine [40]. HA derived from fish bone was reported to be biocompatible [23], and a biomaterial that is not expected to exhibit any cytotoxic effect on dental pulp cells, including dental pulp stem cells, if incorporated into restorative material.

## 5. Conclusions

This study showed that the adding of reinforcements to the powders of Fuji IX and Fuji II in the form of micro-HA particles derived from cuttlefish bone did not improve the CS, DTS, and FS of chemically-set Fuji IX groups, but it improved mechanical properties of Fuji II groups, and with 10 wt% HA, there was a significant improvement of FS.

- Highly porous HA was prepared from aragonitic cuttlefish bone;
- There were four groups for each material: One group without HA particles added and three groups where the powders were modified by incorporation of 2, 5, and 10 wt% HA, respectively;
- Porous HA incorporated into the Fuji IX groups had a negative effect on CS, DTS, and FS;
- CS was significantly reduced in Fuji IX 2 wt% HA and Fuji IX 10 wt% HA groups, and FS in Fuji IX 5 wt% HA, compared to the Fuji IX group without HA particles added;
- The addition of HA particles in Fuji II had a positive impact on CS, DTS, and FS. Fuji II groups modified with 10 wt% HA showed the most favorable results with respect to FS.

## Figures and Tables

**Table 1:** Descriptive statistics for compressive strength in MPa (sample size, mean value, standard deviation, lower and upper bounds for 95% confidence interval.

Material	N	Mean	Standard Deviation	95% CI Lower Bound	95% CI Upper Bound
Fuji II 2	16	153.5	36.1	134.3	172.7
Fuji II 5	17	158.3	25.5	145.2	171.4
Fuji II 10	16	149.2	21.9	137.5	160.8
Fuji II 0	16	141.5	26.5	127.4	155.6
Fuji IX 2	17	80.4	31.6	64.1	96.6
Fuji IX 5	18	92.3	37.4	73.6	110.9
Fuji IX 10	16	82.8	20.9	71.7	93.9
Fuji IX 0	18	111.3	31.2	95.8	126.8

**Table 2:** Descriptive statistics for DTS in MPa (sample size, mean value, standard deviation, lower and upper bounds for 95% confidence interval).

Group	N	Mean	Standard Deviation	95% CI Lower Bound	95% CI Upper Bound
Fuji II 2	15	13.8	3.7	11.7	15.8
Fuji II 5	16	14.3	2.6	12.9	15.7
Fuji II 10	16	12.3	3.1	10.6	14.0
Fuji II 0	16	12.3	4.0	10.1	14.4
Fuji IX 2	18	5.8	2.0	4.8	6.8
Fuji IX 5	17	5.4	2.3	4.2	6.6
Fuji IX 10	16	3.7	1.7	2.8	4.5
Fuji IX 0	15	5.5	1.8	4.5	6.5

**Table 3:** Descriptive statistics for FS in MPa (sample size, mean value, standard deviation, lower and upper bounds for 95% confidence interval).

Material	N	Mean	Standard Deviation	95% CI Lower Bound	95% CI Upper Bound
Fuji II 2	17	41.0	7.7	37.1	45.0
Fuji II 5	17	41.4	8.4	37.1	45.7
Fuji II 10	16	48.4	8.7	43.7	53.0
Fuji II 0	16	36.3	10.8	30.5	42.0
Fuji IX 2	11	12.9	3.1	10.8	15.0
Fuji IX 5	14	11.0	3.0	9.3	12.7
Fuji IX 10	16	13.7	3.4	11.9	15.5
Fuji IX 0	12	15.6	5.0	12.4	18.7

**Table 4:** Result of two-way ANOVA test.

Factor	CS	DTS	FS
Material	<0.0001	<0.0001	<0.0001
HA	0.38	0.06	0.14
Material * HA	0.01	0.15	0.0003

* Stands for interaction. This is a symbol used in two-way ANOVA. The table lists how different material, addition of HA, and the combination of the two (material and HA addition) affect three mechanical properties (CS, DTS and FS).

**Table 5:** Results of the ANOVA test for Fuji II.

Material	CS	DTS	FS	
Fuji II 2	153.5	13.8	41.0	-
Fuji II 5	158.3	14.3	41.4	-
Fuji II 10	149.2	12.3	48.4	a
Fuji II 0	141.5	12.3	36.3	a
*p* *	0.37	-	0.004	-

* *p*-value for ANOVA test; a—materials with the same letter are significantly different (Tukey test).

**Table 6:** Results of the ANOVA test for Fuji IX.

Material	CS		DTS	FS	
Fuji IX 2	80.4	a	5.8	12.9	-
Fuji IX 5	92.3	-	5.4	11.0	a
Fuji IX 10	82.8	b	3.7	13.7	-
Fuji IX 0	111.3	ab	5.5	15.6	a
*p* *	0.019	-	-	0.023	-

* *p*-value for ANOVA test; a, b—materials with the same letter are significantly different (Tukey test).
